# Scent in the Context of Live Performance

**DOI:** 10.1177/2041669520985537

**Published:** 2021-02-03

**Authors:** Charles Spence

**Affiliations:** Crossmodal Research Laboratory, Department of Experimental Psychology, University of Oxford, Oxford, United Kingdom

**Keywords:** scent, theatre, live performance, malodour

## Abstract

Ambient smell has long been a feature of live performance, no matter whether its presence was intentional or not. While, once upon a time, the incidental presence of malodour was an inevitable feature of proceedings, the deliberate use of scent can actually be traced all the way back to the earliest rituals. This review attempts to trace the long history of scent’s use in processions, pageants, and, most important, performance. From Shakespeare’s time onward, scent has been used as an atmospheric/ambient cue. It has been used to create a certain mood, to trigger memories/nostalgia, and, on occasion, it has also served a narrative role. While the use of scent has often been merely illustrative (or pleonastic), there have been numerous occasions where olfactory stimulation has taken on a far more important evaluative role, critical or otherwise. Most often, this has been in the theatre, but also on occasion in the context of the opera, musical, ballet, and comedy too. In recent years, there has been a resurgence of interest in scenting live performance/entertainment, especially in the context of highly immersive and experiential multisensory events. While high-tech solutions to scent delivery have been a prominent feature of its use in the cinema, low-tech solutions have more often been incorporated in the live-performance setting. This and a number of other important differences between scent’s use in the theatrical versus cinematic setting are highlighted.

There has always been an olfactory element to live performance though, to begin with, this was typically incidental. At the same time, however, there has been growing interest in the deliberate use of olfaction in an entertainment context (Banes, 2001), with some commentators talking of the emergence of the theatrical scent designer. Elsewhere, Blankenship (2016) writes, “Let’s call it ‘aroma-turgy’—the conscious use of smell as a part of theatrical design.” While the deliberate incorporation of scent has had a brief and troubled relationship with cinema (see Gilbert, 2008; Spence, 2020c, for reviews), it has had a rather more successful, not to mention much longer, role in the context of live performance—primarily theatre, though, as we will see later, also occasionally in ballet, musical, dance, and opera too. That said, I have yet to come across any mention of scent being used in the music halls or vaudeville shows that were so popular around the opening years of the 20th century (e.g., Arce, 1979; Jackson, 2019). And while the entertainment potential of a scented accompaniment in the setting of the theatre and cinema might, at face value at least, appear similar, it is important to recognize that the approaches, and the media, are fundamentally different, as will be discussed later. This may well, then, help to explain why it is that the incorporation of a scented element into proceedings has been so much more successful in the theatre (as I hope to show later) than in the cinema (see Spence, 2020c, for a review).

## Why Has Scent Become So Popular in a Live-Performance Setting?

According to Dugan (2008), “we have dramatically underestimated the ways in which the staging of smell mattered in the past” (p. 234). Meanwhile, in her hugely influential review, Sally Banes (2001) writes, “The question arises as to why smell has returned to the theatre with a vengeance at the turn of the 21st century” (p. 74). More recently, Blankenship (2016) has written of scented performances that “Several current productions are employing it right now, and their examples underscore both the rewards and frustrations of tickling an audience member’s nose.” On one hand, the recent resurgence of scent in theatre can be seen as hinting at an attempt to compete with movies and television (see Banes, 2001). At the same time, however, there has also been increasing interest in scent in the setting of the art gallery/museum (see Spence, 2020b, for a review), as well as in a musical context (Di Stefano et al., in press; Spence, 2020c). Alternatively, however, it might also reflect nothing more than a general resurgence of interest in the sense of smell (e.g., Henshaw et al., 2018; McGann, 2017).

What is, though, not in any doubt is the growing interest in the role of olfaction in performance studies—for example, see the special issue of *Performance Research* from 2003 (Christie & Gough, 2003). The role that science might play in helping to ensure that scent has the desired effect in a live-performance setting has also intrigued a number of practitioners (e.g., Hill & Paris, 2003; McGinley & McGinley, 2018; cf. Ross, 1938). Of course, the deliberate incorporation of an olfactory element fits right in when it comes to the increasingly popular move towards more experiential/immersive entertainment, across many media.^1^

That being said, it is also important to note that not everyone is convinced that scent is deliberately being used in a theatrical context. For instance, according to Jon Whitmore (1994),

The engagement of the audience’s senses of smell, touch, and taste plays a secondary role in most traditional theatre productions. This is an interesting phenomenon, because these three senses are the only three that cannot be employed in any meaningful way by the movies or television. (p. 201)

Meanwhile, writing a few years later, Kirsten Shepherd-Barr (1999, p. 152) made much the same point, when writing of Roinard’s 1891 production of the *Cantique des cantiques* (about which, more later) that it “stands as a unique example of the use of smell in theatre; only a few such experiments have ever been made.”

## Scent in a Live-Performance Setting: Theatre, Musical, Opera, Vaudeville, and Music Hall

The majority of the press interest in scented entertainment has tended to focus on the technological release/control of the context of cinema (e.g., see the extensive coverage in Gilbert’s, 2008 book, *What the Nose Knows*; and Spence, 2020c, for a recent review). There has been parallel interest in scenting the theatre, though, as we will see later, the challenges and opportunities in the context of live performance are very different from those of the plastic arts (e.g., Henshaw et al., 2018).

## Review Outline

This critical narrative review aims to provide a chronological assessment of the various ways in which ambient scents/smells have impacted the multisensory experiences of Western audiences in a live-performance setting from ancient times through to the present day.^2^ The focus is primarily on the use of ambient scent in an entertainment (i.e., rather than a ritualistic) context. From the very beginning, there has been an olfactory element to live performance. There is, after all, no getting away from the fact that people smelled, as did the enclosed public and private spaces that they inhabited. Or, as David Potter (1999) once put it: “There can be no question but that the urban air of the Roman empire stank” (p. 169). Public gatherings, such as theatrical performances and other forms of public performance, were likely to have been particularly smelly encounters (e.g., D’Errico, 2018, p. 228). In the next section, I highlight the early problem of malodour and some of the scented steps that have been taken to address the problem.

While initially the incorporation of scent may have been nothing more than incidental as when actors would cook, smoke, or apply perfume on stage, the realization soon emerged that scent could be used to enhance the realism of a production for the audience (Blankenship, 2016). As F. T. Marinetti (1971) notes,

The Variety Theatre uses the smoke of cigars and cigarettes to join the atmosphere of the theatre to that of the stage. And because the audience cooperates in this way with the actors’ fantasy, the action develops simultaneously on the stage, in the boxes, and in the orchestra. (p. 118)

In the “On the Deliberate Use of Scent Since the End of the 19th Century” section, I summarize the various occasions in which a scented element has, over the years, been introduced (either incidentally or deliberately) into a live-performance setting, focusing primarily on the theatrical context.

Thereafter, in the “Problems With Bringing Scent to a Live-Performance Setting” section, I take a closer look at the question of whether deliberately scenting entertainment can ever be anything more than a “gimmick”—one of the criticisms, note, that has often been levelled at the use of scent in cinema (see Spence, 2020a, for a review). The various methods used to deliver scent in a live-performance setting are also discussed, as well as problems related to audience members misinterpreting the meaning of (often synthetic) scents. Along the way, I will highlight a number of the key differences between the use of scent in the theatre and cinema, hence perhaps helping to explain why the deliberate incorporation of scent has been rather more successful in one context than the other. In the final section, I summarize the multitude of ways in which scent has, and is currently, being incorporated in an ever more central manner into a wide variety of live-performance productions.

## On the Early Use/Presence of Scent/Malodour in Live-Performance Setting

The olfactory element of divine rituals (Classen et al., 1994) was sometimes incorporated into early plays. According to Banes (2001),

The beginnings of Western theatre in ancient Greek festivals like the Eleusinian mysteries (in modern times considered the prototype of the modern gesamtkunstwerk) were suffused with intense aromas of all kinds—including fruit, floral, grain, and animal offerings; blood and burning animal flesh; wine, honey, and oil libations; and the burning of incense and other materials in sacred fires (see Burkert, 1985). (p. 68)

To this list of odours, Alipaz (2015) adds the smell of burning herbs, noting that Roman theatres were regularly scented with saffron to mask the ambient (mal-)odour (cf. Bradley, 2014; Clements, 2013).

According to Bradley (2015), “Greek drama was typically staged in the context of religious festivals, and some plays exploited the olfactory experiences of sacrifice, smoke, libations and feasting into their content” (p. 2). Bradley continues:

From the Hellenistic period onward, scents and perfume were sometimes integrated into dramatic performances, and writers such as Ovid and Apuleius describe the ingenuities of engineering in contemporary theatre which might include features like a fine saffron mist sprayed over the audience.

As has seemingly always been the case, there have been critics of scent’s use in an entertainment context, according to one:

Such dramatic developments had their critics: Aristotle and Horace, among others, denounced the sensationalism of contemporary theatre, which distracted the audience from what really mattered … and which contributed nothing to the accurate understanding of the events that were unfolding before their eyes and ears (Aristotle, Poetics 6.28; Horace, Epistles 2.182-207).

Smell also played an important role in the early comedies of Aristophanes and Cratinus. Although, in the latter case, it was more verbal humour about bad smells than necessarily the delivery of specific scents that the audience could themselves inhale (see Telò, 2013). Meanwhile, of entertainment in ancient Rome, Koloski-Ostrow (2015) suggests that

While the plays, slapstick skits and readings held in theatres did not have occasion to raise the stink of the ampitheatres, the sale of cheap foodstuffs and the sweat of performances held in the heat of the morning generated powerful smells. The oldest types of stage show were apparently quite vulgar, displaying nudity, violence, sex and the real or imagined smells from loud burps and louder bursts of flatulence. (p. 105)

### Scented Processions and Pageantry

According to Dugan (2008),

Scents, as stage properties, fulfilled a host of practical purposes, fusing crowd control, religious symbolism, monarchical power, civic procession, and mercantile interests with thematic content in a wide variety of dramatic performances. When read within the context of late medieval and early modern English stage history, these descriptions reveal that we have dramatically underestimated the ways in which the staging of smell mattered in the past. (pp. 223–224)

Dugan (2008, p. 231) goes on to highlight how formal processions and pageantry would sometimes have incorporated a scented element into the proceedings. For instance, she mentions how rosewater “fumigations” were used to protect Edward IV’s queen, Elizabeth Woodville from the stench of the crowd during the 1,464 pageants celebrating her arrival in London (as documented in Wickham, 2002, p. 96).

Dugan (2008) also notes how aromatic rain and scented “hail” was incorporated in the York Pageant of Our Lady of 1486. There are also records of incense being used for various revels at Edinburgh Castle in the 1470s and 1480s (Mill, 1969, p. 57), and scented fountains were said to have provided an ambient setting for an interlude designed for Henry VIII in honour of the French ambassador. Dugan (2008, p. 232) notes how a similar fountain was thereafter created for one of James I’s coronation pageants in 1603 in London. In fact, mayoral processions in the 16th and 17th centuries used scents to help celebrate London’s civic and economic power in the spice trade (Barbour, 2003, p. 97). According to Dugan (2008, p. 233), Thomas Middleton’s *Triumphs of Honour and Industry* in 1617, and the *Triumphs of Honour and Virtue* in 1622, both involved elaborate staging of *“all manners”* of *“spice plants and trees bearing odour*.*”*

Dugan (2008) also writes that

Perfumes were also employed in Elizabeth’s courtly revels. As account records from the Office of the Revels document, numerous performances in the early years of her reign utilized bushels of aromatic herbs, roses, and vegetation — either strewn about the stage floor or construed into elaborate scenery (Feuillerat, 1908). In 1572, Robert Moorer, an apothecary, provided musk, ginger, and clove comfits, along with quarts of rose water and pints of spike water, to create scented flakes of ice and hail in the “Masque of the Janus” presented to the queen. He also supplied similar ingredients for the “Masque of the Wildman,” performed at court on Twelfth Night, 1573 (Feuillerat, 1908). (p. 233)

Before moving on, it is perhaps also worth making the point here that not all examples of public scenting in premodern performance venues should necessarily be framed in terms of attempts to mitigate the malodour of the masses. As an example, Classen et al. (1994, p. 27) note that corporate, shared perfume wearing at Roman public events served values of solidarity, writing that “Putting on a good show in antiquity … involved putting out a good scent. . . .Not only would the spectators see and hear the pageantry, they would breathe it in and feel identified with it and each other.” There are also values of hospitality around scent sharing that constitute an important part of Middle Eastern culture too. Unfortunately, however, reviewing the cross-cultural perspective on the shared experience of scent falls out of the scope of the review.

### Scented Theatre in the 17th Century

Harris (2007, p. 484) notes that the stage directions for two of the plays performed by *The King’s Men* in 1603 and 1613 included instructions that called for the burning of incense. Dugan (2008, p. 230) provides a number of examples of scent’s frequent presence in the text,^3^ and possibly also in the theatre of Late Medieval and Early Modern England in what she describes as “Three remarkably odiferous plays … the Digby Mary Magdalene and Shakespeare’s Antony and Cleopatra and Twelfth Night.”

One of Shakespeare’s other plays, *Macbeth*, first performed in 1606, opens with the stage directions calling for thunder and lightning. These would have been recreated by means of the detonation of squibs, small fireworks, which would have filled the theatre with the smell of gunpowder, and thus provided playgoers with an olfactory reference for the “three witches” bizarre incantation: “Hover through the fog and filthy air” (Harris, 2007, p. 465). The sulphurous stink would have persisted through the first scene as the firework’s thick smoke slowly wafted across the stage and into the audience. The association between a sulphurous bad smell and Hell has been traced back to the Corpus Christi drama (Mills, 1992, pp. 302–303), the annual play that was presented by the Chester Cooks. This production involved a hell mouth that may have emitted smoke and possibly also a terrible stench.

According to Harris (2007), audiences watching *Macbeth* in the early 1600s would likely have connected the pervasive presence of the sulphurous smell of gunpowder in the theatre, both to the on-stage events as well as to recent political events in England, namely the plot by Guy Fawkes and his Catholic coconspirators to blow up Parliament. The Gunpowder Plot had been uncovered in the months preceding the play’s first performance. Harris also raises the possibility that the detonation of the noisy smelly squibs might have been connected with the celebratory use of fireworks. The polychronous associations of the sulphurous olfactory atmosphere in this Shakespearian play likely triggered multiple memories in the audiences of the day (see Harris, 2007, p. 473; see also Gurr, 2004), thus going well beyond the merely pleonastic use of scent to redundantly show what was on the stage (about which, more later; see Banes, 2001).

### Tackling Malodour in the Theatre

One of the principal uses of scent in early performance may well have been simply to help mask the ambient malodour. According to Harris (2007), in Shakespeare’s time, the theatre likely smelled terrible. People would often bring their own food and drink to the performance. As such, there would have been a variety of unintended smells including everything from what people were eating through to the fetid odour emanating from the urine trough (Banes, 2001).^4^ Meanwhile, according to Alipaz (2015), the travelling shows of the late renaissance and early baroque periods, called commedia dell’arte, would have been performed in carnivals, markets, and dining halls, with all of the attendant smells. In terms of the olfactory atmosphere at the opera, a live-performance format that was introduced during the high baroque, Alipaz conjures up the smell of burning candles, sweaty crowds in smelly costumes, and the smell of the eggs used to bind the makeup. All of this, once again, would have been accompanied by the odours associated with the food and drink that was commonly consumed during long performances.

While commentators nowadays are often horrified by the malodour that they imagine must have assailed the nostrils of those trying to enjoy the live performances of centuries gone by, it is important to note that things might have smelled different to our forebears (Smith, 2007). Indeed, it is not altogether certain that, at the time, people would necessarily have had anything like the same aversive response to ubiquitous everyday malodour (e.g., of body odour and toilet smells). As such, it is at least worth questioning whether, being an ever-present and unavoidable aspect of the olfactory backdrop, the ambient malodour would be effectively ignored, or else people would have adapted to it. Intriguing in this regard are Roman urban commentators who complain about the smell of the countryside not the undoubtedly malodourous city (e.g., see Morley, 2015, p. 117).

The tradition of men wearing gardenias in their lapels would presumably also have helped to mask their body odour (e.g., Bussell, n.d.; Sebag-Montefiore, 2016). Perfume also became cheaper, and hence, its use became more widespread in Europe during the 19th century (see Jenner, 2011, p. 340). Audiences at the end of the 19th century would have been more likely to have enhanced personal hygiene, given the growing emphasis on deodorization.^5^ Nevertheless, fragrant fountains were, on occasion, also to be found in the lobbies of the theatres. Meanwhile, fragranced programme fans were occasionally featured at theatrical performances in the latter half of the 19th century (e.g., in London; Banes, 2001, p. 68; Haill, 1987; Hawking, 2015). Eugène Rimmel, the French-born, London-based perfumer, famously provided scent fountains in the lobbies as well as souvenir printed programme fans for the opening of the Gaiety Theatre, Strand, 21 December 1868 (Alipaz, 2015; “Fan – Rimmel’s programme fan,” n.d.). The scented theatre programmes that were handed out at performances were often fragranced with one of Rimmel’s favourite perfumes, “Royal Aquarium Bouquet” (Alipaz, 2015). The scent would have permeated the entire printed programme, thus presumably acting as an effective advertisement for Rimmel and his wares.

The prolific perfumer also produced a Vaporizer, advertised in the middle of 19th century (see Rimmel, 1865), as fragrancing the air at seven different entertainment houses (including Her Majesty’s Theatre, St. James’s Hall, Lyceum Theatre, Hanover Square Rooms, Mr. & Mrs. Howard Paul’s Entertainment, Mr. Woodin’s Cabinet of Curiosities), as well as on board the H. R. H. Prince of Wales royal steam yacht (see Lambert, 2013). The scented fans, fountains, and vaporizers may therefore have helped to mask the malodour prior to the widespread adoption of modern hygiene practices. However, intriguing though such examples are, it is worth remembering that the scenting of theatres would have been a relatively rare occurrence and essentially restricted just to London theatres.

Intriguingly, however, from 1864 onward, Rimmel’s scents start to be incorporated as integral part of scenes on stage, and not just as a means of masking the malodour (Alipaz, 2015). For instance, an 1873 playbill for *Antony and Cleopatra* at Drury Lane suggests that one scene incorporates perfume, stating that “The perfume is produced by means of Rimmel’s persian ribbon.” Meanwhile, another of the playbills unearthed by Alipaz for a production of *Lady Belle Belle*, Scene 4, “The orange Grove” at the Adelphi Theater reads: “In this scene the perfume of ‘Winter Flowers’ will be introduced by Rimmel’s Vaporizer.” These examples, note, all seem to fit as the ambient/atmospheric use of fragrant rather than narrative (see later, for more on this distinction; Banes, 2001).

## On the Deliberate Use of Scent Since the End of the 19th Century

The use of scent in the context of live performance certainly picks up in the closing years of the 19th century. It is, after all, perhaps at around this time that the smell of the audience (their food, their body odour, and their effluence) may no longer have been so much of a concern, given improved sanitation and personal hygiene (Jenner, 2011; Stamp, 2014). Hence, the deliberate use of scent would presumably have had more of a chance of reaching the audience’s nostrils and could thus be used for its ambient (atmospheric) and/or narrative effect, rather than merely to mask the malodour of the masses, as had perhaps so often been the case before.

### Symbolist Performances

Several Symbolist artistic performances took place around the end of the 19th century. They typically involved audiences being presented with various combinations of colours, sounds, and scents that were thought to correspond with one another (see Fleischer, 2007; Marvick, 1999; Shepherd-Barr, 1999). In one much-discussed early example, Paul-Napoléon Roinard delivered two performances of the Old Testament text of the *Cantique des cantiques* (Song of Songs) of Solomon at the Théâtre d'Art's in Paris on the 10 and 11 December 1891. Roinard apparently had in mind an almost synaesthetic multisensory work in which an original musical score, words, vowel sounds, colours, and scents were all to be harmonized (Halperin, 1988, p. 199; Stokes, 1972, p. 167). Intriguingly, Roinard provided details of the specific combinations of scent (e.g., “frankincense”), colour (e.g., “pale purple”), speech (e.g., “in i illuminated with o (white)”), and music (e.g., “in C”) that were to be presented in synchrony during each one of the eight poetic sections, or “mystic devices” (see Fleischer, 2007, p. 111).

A total of nine scents (frankincense, white violets, hyacinth, lilies, acacia, lily of the valley, syringa, orange blossom, and jasmine)^6^ were released sequentially into the theatre while the audience listened to words and music (Roinard et al., 1976). According to Shepherd-Barr (1999), the scents were diffused by young Symbolist poets, being “pumped in” (p. 152) by those stationed on the far edges of the proscenium, while being dispersed using handheld vaporizers by those situated in the balcony (Deak, 1993, p. 155). While Roinard had wanted to realize the ideal of a synthetic and perfect union of the arts and of the senses, practical constraints with delivering, and thereafter rapidly clearing, so many scents may well have limited the audience’s appreciation of this work (e.g., Deak, 1993, p. 156; Fleischer, 2007; Shepherd-Barr, 1999).^7^ More fundamentally, though, one can also question whether the specific crossmodal mappings that Roinard suggested really did correspond crossmodally (i.e., in a manner that would have been meaningful to the majority of the audience), or whether instead they may have been based on nothing more than idiosyncratic (possibly synaesthetic) combinations of stimuli instead (see Spence, 2020c, on this theme).

A subsequent Symbolist dance performance inspired by Baudelaire’s poetry (see Baudelaire, 1958) and Roinard’s Symbolist staging of *The Song of Songs* took place in Paris in 1913. In Valentine de St.-Point’s “metachorie,” large pots of incense were burned, according to the dancer’s theory of the correspondences governing the scent, predominant colour, musical environment, and central poetic idea associated with each dance (Moore, 1997; cf. Banes, 2001). In this case, though, I am not aware of any record of the specific crossmodal correspondences that were incorporated into the work and whether they would have been any more meaningful to the audiences of the day than those that had been presented by Roinard some 22 years earlier. Nevertheless, it would appear that the Symbolist’s attempts to connect diverse unisensory impressions (namely scents, sights, and sounds) in a manner that would resonate with audiences turned out to be rather more difficult to achieve than it may at first have seemed to those concerned. This growing realization would, one presumes, have dampened the enthusiasm of those artists who were tempted to meddle in this space of scented Symbolist performance.^8^

### Olfactory Storytelling

On 30 November 1902, Carl Sadakichi Hartmann incorporated a series of semantically meaningful scents into his performance of *A Trip to Japan in Sixteen Minutes* presented at the New York Theatre. According to Hartmann’s (1913) description, he read out a text while a pair of geishas danced on stage. The one and only performance of this work was accompanied by the sequential release of a series of eight scents. In this case, Hartmann describes how he chose scents that would be perceptible when presented one after another. Hartmann’s aim with this work was to evoke the stages (and locations) of the journey by means of the semantically meaningful scents associated with specific places, including the smell of almond for Southern France, bergamot for Italy, cedarwood for India, and carnation to represent Japan. Hartmann had assumed that the scents would be recognized by the audience, hence triggering thoughts on the appropriate (i.e., intended) theme/place. However, anecdotal reports suggested instead that people’s emotional associations with these scents tended to be somewhat unpredictable, as Hartmann himself was forced to admit, when writing about the performance some years later (see Hartmann, 1913, p. 222).^9^ Once again, this one-off experience was widely regarded as a critical failure (see Hartmann, 1913; Shepherd-Barr, 1999). Technical problems with the release and clearance of the various scents were partly to blame.^10^ At the same time, however, it is also possible that the audience of the day may simply not have been ready to appreciate the innovative scent-based approach to storytelling that Hartmann had in mind. Later in this review, though, we will see how others have recently started to revive the art of olfactory storytelling (e.g., see Delaunay, 2014).

### On the Ambient, Atmospheric, and Narrative Use of Scent

Given the failure of both the Symbolist and olfactory storytelling approaches, it should perhaps come as little surprise to find that the majority of (successful) cases in which scent has been incorporated in a theatre/live-performance setting from the late 19th century onward have all tended to involve the ambient (atmospheric), and/or the narrative, use of scent. For example, theatre scent designer David Bernstein had the following to say:

I split up scent design into two categories. One is an ambient smell or scent, which is scenting the theatre or the performance space as the audience is walking in. It’s part of the initial impression. It’s more like an installation, so it serves to transport the setting, or to make it “other.” The second is more like scent cues. Rather than scenting the space when you walk in, it’s the introduction of aromas to coincide with the action on stage. (Slagle, 2014)^11^

The examples of the use of scent in a live-performance setting that we will come across below have certainly tended to be much more austere, both in terms of the number of scents introduced during a performance (typically, just a single atmospheric scent has been released), and in terms of the scope of what scent is supposed to deliver in terms of the audience’s multisensory experience. The majority of other early uses of scent in the theatre/live-performance context have tended to involve a very literal matching of the scent to the scene and/or action portrayed on stage. It is easy to imagine how the latter, redundant, approach to incorporating scent would, at least presumably, have helped to increase the likelihood of the appropriate associations being triggered in the audience’s mind. That said, Banes (2001) is critical of this kind of approach, writing at one point that “ … so often the use of smell seems merely iconic and illustrative, a weak link in a chain of redundancy across sensory channels that does nothing more than repeat what is already available visually and aurally” (pp. 68–69) (cf. Feagin, 2018). Rather than merely just using smell to illustrate what can be seen on stage, its incorporation is more interesting when used to induce a particular mood or atmosphere.

The incorporation of a single atmospheric (or ambient) scent into theatrical proceedings has been used on many occasions (and hence, in many different productions) over the past 130 years or so. One of the first documented uses in a live-performance setting was in André Antoine’s 1888 production of Fernand Icres’s *The Butchers*. According to Banes (2001), several olfactorily-redolent sides of beef were used as stage props in this particular production. It is, though, unclear whether the meaty odour of the carcasses in this case was anything more than an accidental, and presumably unavoidable, consequence (or by-product) of this particular choice of realistic stage prop. The same cannot be said of the Chinese incense that was burned in *The First Born* (1897), a play set in San Francisco’s Chinatown (Marker, 1974, pp. 61–63). Nevertheless, both of these early examples can be framed in terms of helping to set the multisensory scene by providing an olfactory context that would have been unambiguously congruent, and hence redundant, with the setting on stage. In December 1891, the same month as Roinard’s production, Maeterlinck’s *The Blind* was also performed in Paris, by the same company “in a theatre sprayed with perfume to make it more atmospheric” (Slater, 1997, p. xiv).

Moving beyond the use of ambient scent merely to match the stage setting, others have been interested in using it to help set the mood or ambience. For instance, proscenium servants sprayed luxurious perfume into the auditorium to create a sense of luxury in Vsevolod Meyerhold’s 1910 production of the opera *Don Juan* (*Don Giovanni*; Leach, 1989, pp. 89–90). This more abstract use of scent was, though, something of an exception in the early days of scent-enhanced entertainment (though see Banes, 2001). Far more frequently, it can be argued, scent has been used simply to redundantly help set the scene, as, for example, when director David Belasco (1853–1931; http://www.valentinetheatre.com/mural/bios/David_Belasco.html; see Anonymous, 2014) had a children’s restaurant on stage complete with the aroma of pancakes, which were prepared during the play *The Governor’s Lady* (1912). In another production, Belasco had his actors make coffee on stage so that the aroma would spread through the auditorium to enhance the realism of the production (http://www.valentinetheatre.com/mural/bios/David_Belasco.html). Meanwhile, according to Banes (2001), pine needles were scattered across the stage floor to create an appropriate scent for the forest setting in the 1917 production of *Tiger Rose*.

Elsewhere, the scent of orange was released in the theatre while the song “An orange grove in California” was performed in the 1923 *Music Box Revue* (Crowther, 1959). There was also a lilac-scented accompaniment to *Lilac Time*, by George Fitzmaurice in 1929 at Boston’s Fenway Theatre (Gilbert, 2008, p. 149), while *The Broadway Melody* (1929) by Harry Beaumont (incidentally, the first proper musical) incorporated an orange scent that was sprayed into the auditorium (Sobchack, 2013). A rose perfume was released to coincide with the Persian Garden scene of the 1952 Paris Opera revival of Rameau’s opera-ballet *Les Indes Galantes* (Guest, 1976, p. 201), while the smell of perfume apparently wafted from the silken canopies of the set in Graeme Murphy’s ballet Shhe'razade performed by the Sydney Dance Company in 1979 (Cargher, 1979, p. 47).

The audience’s experience of a scented element to theatrical proceedings has very often been merely incidental, for example, as when the scent of actor’s smoking or cooking on stage can be experienced olfactorily by the audience (Margolies, 2003). At the same time, however, when the audience actually smells the performer and/or their action/performance, this awareness can nevertheless still help to integrate the former into the action and may have a spatial bodily/perspectival component (Feagin, 2018; see also Clapp, 2000; McGinley & McGinley, 2018; McMillan, 1999; Reason, 2003; Reinarz, 2018). According to one review of Het Zuidelijk Toneel’s use of scent in *India Song*, the commentator described the audience as “bathed in unexpected and intense smells and sounds” (McMillan, 1999). By contrast, of course, when scent is added to cinema, it is obvious that it is not the same scent that the actors seen on screen are experiencing, thus, in some sense, serving to distance the audience from the on-screen action instead.

According to Holly Dugan (2009), smell helps to connect “the environment of the theater with the dramatic world unfolding on stage” (p. 733). Carolyn Sale (2006, p. 145) has also emphasized the material connection that is created between actors and audience, both of whom, in her words, “eat” the same air and “feel” the same smells. The members of the audience in Renegade Theater’s 2015 production of *Glass: Shattered*, an adaptation of Tennessee William’s *The Glass Menagerie*, smelled the perfume shortly after the actress playing Amanda had sprayed it on herself prior to going on stage. Feagin (2018) writes:

For example, in Glass Shattered, when I smelled Amanda’s perfume, I instantly felt myself to be in the same space she was in, and in the same room with her, the same place. Further, this awareness of my own bodily presence was relevant to understanding and appreciating the performance. Several aspects of the production were designed to break down the ordinary theatrical conventions that the audience and characters occupy different spaces and places.^12^ (p. 137)

According to an anonymous reply to an intriguing online article that appeared in *Frangrantica* by Alipaz (2015), the actress Alla Demidova once wrote how the actor Vladimir Vysotsky brought bottles of Parisian perfume to spray on them before performing (as Ranevskaya and Lopakhin, respectively) in *The Cherry Orchard*.

Over the last half century or so, though, the use of scent has often become rather more sophisticated, its use often intended to convey a more pointed, political, or critical/evaluative message. In such cases, note, olfaction’s incorporation into theatrical proceedings starts to go beyond the merely incidental (Feagin, 2018; Margolies, 2003), illustrative, atmospheric (or mood-inducing), or straightforwardly narrative (see Banes, 2001). In this regard, one might, for instance, want to consider the smell of marijuana that was apparently often to be smelled during performances of the 1969 musical “HAIR” (Wittek, 1998). Likely to have been rather more jarring for the audience’s nostrils, though, was the smell of the large number of cleaning products that were deliberately introduced during Shaun Lynch’s *Clean Smell Opera* (1980). The aim, in the latter case, was for the smell to eventually become overpowering, and hence repugnant, to the audience (Carroll, 1980). Philip Prowse’s *Tis Pity She’s a Whore*, at Glasgow Citizen’s Theatre in 1983, had a “sort of charnel-house stench of death and incense which hung over whole production.” On this occasion, according to Hawking (2015), the smell was meant to signal “the contrast between the sickness of the incestuous love, and the suffocating formal atmosphere.” Meanwhile, various “unhygienic” 18th century odours were incorporated in Mark Wing-Davey’s 1995 production of *The Beaux' Stratagem*, a play set in London in the opening years of the 18th century (Winn, 1995, p. 35).

The strawberry-scented room spray in the 1996 New York production of Joe Orton’s Entertaining Mr. Sloane, directed by David Esbjornson, was used to create an intentionally tacky ambience. As *The New York Times* theatre critic Ben Brantley described it,

It isn’t what most people would think of as a sexually tantalizing smell: floral, fruity and unquestionably synthetic, it is as welcome to the nostrils as a vinyl handkerchief … Smell, thank goodness, is not a sense that’s much exploited in the theater. But when Ms. Parker’s strawberry spray wafts into the audience at the Classic Stage Company, it feels ingeniously apt, an aromatic equivalent of what you've been hearing on stage. Orton’s characters do indeed seem to speak the verbal equivalent of cheap air freshener. (Brantley, 1996, p. 22)

Other scented productions include Ivo van Hove’s 1999 *India Song* where the smells of manure, diesel, and citronella were introduced, though what the intention behind this use of scent was, I have not been able to ascertain (Wilson, 1999, p. 8). According to Banes (2001, p. 72), the smell of fish and chips was a key element of the 1999 New York production of Ayub Khan-Din’s play *East is East*, a bittersweet comedy about an Anglo-Pakistani working-class family in England in the 1970s. Banes highlights how the two published reviews of the play start by discussing the distinctive odour that pervades the theatre before the play begins (see Brantley, 1999, p. C13; Kuchwara, 1999, p. D3).^13^ And, arguing against the common suggestion that we tend to rapidly adapt to ambient smell, Ben Brantley starts his review of the play by saying:

The smell is what you notice first, even before you’ve taken a proper look at the stage from which it emanates. It’s the thick aroma of fat heated up for deep frying, mixed with fleeting suggestions of spice, and you can’t shake it out of your nostrils for the rest of the evening.

Meanwhile, David Bernstein, one of foremost contemporary proponents of “scent design” in theatre (that, according to Hawking, 2015) introduced the smell of “Old Spice deodorant, crayons, condensed milk and a riding crop” to a scene in which the protagonist loses her virginity in the 2010 production, *You Are Made of Stars*. More recently, a convection oven and an overly cinnamon-and-nutmeg-saturated apple pie recipe was used to set—and scent—the Broadway show *Waitress* (Foster, 2017; Paulson, 2016; see also Daniels, 2018). Writing about this case, Feagin (2018) makes much of the fact that the oven itself was situated offstage, and so not visible to the audience, arguing in this case that the use of scent may have helped to modify the audience’s spatial awareness. She writes:

It would be interesting to know if the physical explorations of olfactory curiosity tend to be stronger when the audience can smell something that the characters do not, or when the bully in the sensorium has been temporarily rendered ineffective so that the audience cannot see where the scent is coming from. (Feagin, 2018, p. 143)

A blast of jasmine was incorporated into Katori Hall’s *Our Lady of Kibeho* at the Signature Theatre in New York. That said, the absence of scent in the subsequent 2014 NY production (see Isherwood, 2014) hints perhaps at olfaction’s idiosyncratic/ephemeral use in the live-performance setting. Meanwhile, a “horse-like smell” was incorporated into a scene featuring menacing balrogs in the musical adaptation of *The Lord of the Rings* (Blankenship, 2016).^14^ (Trying to figure out what mythical creatures should smell like is presumably much like the problem of what new devices, like the lightsaber in *Star Wars*, should sound like.)

Live performances have seemingly started to incorporate more scents into their productions in recent decades (e.g., Foster, 2017; McGinley & McGinley, 2018; Reynolds, 1989), though still falling some considerable way behind the 30 plus scents that were sometimes released in theatres in the brief heyday of scented cinema (see Spence, 2020c, for a review). Scratch-and-sniff cards were used in the 1989 production of Prokofiev’s opera, *Love for Three Oranges* (Reynolds, 1989). According to reviews, though, the scents were not presented at moments of dramatic tension but rather when there was lots of noise on stage. As Robert Maycock (1989) puts it, “Musically the performance has an intensity to match the … and carry the horrors of the scratch-and-sniff cards which fill the house with vaguely chemical odours and double the coughing score of an already seasonally bronchitic audience.” In fact, one of the odours was described as smelling something like “a cross between bad eggs and body odour” (Reynolds, 1989).^15^ At the same time, however, there is also a danger that the different sensory inputs may compete for the audience’s attention, and hence, a scent is simply less likely to be noticed if there should happen to be loud noise at the same time (see McGinley & McGinley, 2018, p. 222).

Avery Gilbert (2008, pp. 144–146) also briefly mentions an unsuccessful attempt to bring scent to a theatrical production that he was involved in, back in 1993. Called *Blind Trust,* this performance, put on by the New Opera Theatre Ensemble, took place in complete darkness at the planetarium in the Boston Science Museum. Scents were used to accompany the scenes and give a sense of place, with Gilbert describing how he sourced/created smells that would be appropriate for a pizza parlour, a flower shop, a laundry, and a movie theatre. The scents, which were sprayed from aerosols held by the four air inlets were, however, apparently lost in the large space. Gilbert also highlights the lack of synchronization between the scenes and the scents, with the latter sometimes being released too early. Gilbert suggests that “Instead of building a multisensory realism, the scent effects sowed confusion” (Gilbert, 2008, p. 145). Reviewing the performance, the *Boston Globe* described the odours as “confusing and unpleasant” (as quoted in Gilbert, 2008, p. 146).

*Festen*, the play based on the 1998 Danish movie of the same name, was also put on in Annecy, France followed by performances in Paris and 14 other French cities, opening on 7 November 2017. Three scents created by the perfumer, Francis Kurkdijan, were released at various moments in the play, directed by Cyril Teste. Scent dispersal was achieved in this case by means of modified bladeless Dyson fans. According to Foster (2017), “Mr. Teste hopes to draw people in by their noses, rather than their eyes.” The first scent was described as a wet, mossy forest smell and was dispersed just as the last few members of the audience were taking their seats. The second scent, an ashy, fiery smell was meant to represent a fireplace, after the fire has been extinguished. In this case, the “sour” smell was supposed to capture the tense relationship between the abusing father and his son. The third scent was meant to be the perfume of the daughter who had committed suicide, and the play ended with a return to the initial forest scent.

It is intriguing to note how the use of scent in *Festen* was intended to be symbolic and/or mood/memory-evoking rather than merely replicating in olfactory form what could be seen on stage. Given the fact that the latter play incorporated multiple scents, and performances were also given at a wide range of different venues, one can only imagine how challenging it must have been to try and ensure effective scent delivery (and clearance, about which, more later). At the same time, however, as Feagin (2018) notes, the smaller spaces in which theatrical productions are often put on, including the theatre in the round format (see also McGinley & McGinley, 2018), can make ambient scenting a somewhat less challenging proposition than in the case of a movie theatre, or in the context of opera/ballet, where the venues generally tend to be much larger (Spence, 2020b). One of the challenges is that it can be hard to ensure uniform distribution of scent, as production director Ben Randle noted of one show: “Depending on where you are in the room, you might smell something, or you might not.” Nevertheless, he goes on to say that

As elusive and frustrating as it can be, however, aroma-turgy may still be worth the effort. “It’s going to give a sense of specificity to some people, and for others, it will just trigger an emotional response, … Frankly, I think that’s okay.” (quoted in Blankenship, 2016)

*Treasure Island*, a production at the Theatre in the Round in Minneapolis, Minnesota in April 2014, also included four scents, which according to McGinley and McGinley (2018, p. 224) were the smell of the pirate ship, the old tavern, sea breezes, and Skull Island jungle. Beyond the various theatrical uses of scent that have been described so far in this section, other examples mentioned by Jon Whitmore in his 1994 book *Directing Postmodern Theatre* include by Peter Brook,^16^ The Living Theatre, the Polish Laboratory Theatre, and large-scale musical spectacles such as *Les Misérables* (Whitmore, 1994, p. 195; as quoted in Shepherd-Barr, 1999, p. 158; see also Garner Jr., 2007; McGinley & McGinley, 2018, for a number of other scented productions). The main point to note here is that scent has actually been used far more often in a theatrical context than some commentators would have us believe (e.g., see Shepherd-Barr, 1999, p. 152, as quoted earlier).^17^ That said, the deliberate use of scent in other live-performance contexts such as opera, ballet, dance, or musicals appears to have been rather less common than in the theatrical setting.

### On the Use of Scent in Immersive Theatre

One of the live-performance contexts where scents as well as other sensory cues are especially common is immersive theatre. The popularity of immersive (and hence experiential) theatre has increased dramatically over the past 20 years or so. Secret Cinema has hosted nearly 50 productions in which audience members enter a world mimicking that of a film (Anonymous, 2019). Dalton Dale, the founder of the Space 18 theatre in London, describes it as follows: “I call this 4D theatre. You make the audience hear it, smell it, feel it” (quoted in Anonymous, 2019, p. 17). Immersive theatre often engages additional sensory cues, such as the use of temperature (and temperature change) to convey a different setting or atmosphere to the audience, as, for example, in the New York production of *Sleep no More* by the theatre company Punchdrunk that your author remembers so well (Rose, 2012). Scent is often a distinctive feature of immersive theatre productions too. For instance, the production of *Ghost Ward* at the Almeida involved the audience being bused off to a disused hospital where one critic started her description of the multisensory experience as follows: “The experience is fleeting, fragmentary and mysterious. You walk down a corridor that smells of cabbage … ” (Gardner, 2001).

Jennine Willett’s company, Third Rail Projects, staged an olfactorily-redolent production of *The Grand Paradise* in a warehouse in Brooklyn (Blankenship, 2016). In this fully immersive experience, the members of the audience were dropped off into a fictional beach resort in the 1970s. According to reports, there were actors and objects to interact with in every room. What is more, the olfactory stimuli had been carefully curated throughout. So, for instance, when the audience members encountered a male hustler in a small bedroom, his pillowcase had been deliberately dabbed with vintage cologne. According to Willett, “Maybe it’s three people a night that walk away going, ‘Oh my God, that guy smelled just like my boyfriend in high school.’” He continues: “But I think that’s a win. It’s great when people have a moment to connect to something from a personal space.”

Notice here how the previous quote would appear to mark something of a shift away from the traditional concern about trying to ensure that everyone (or nearly everyone) interprets the smell in the same way (cf. Hartmann, 1913; Runciman, 1915, p. 156; Shepherd-Barr, 1999), to embracing the diversity of responses that the use of ambient scents may well elicit. Note also that such an emotional response does not necessarily require the audience to be able to describe, or identify, the smell itself. This is perhaps just as well, given that people are notoriously bad at doing both of these things (e.g., Majid & Burenhult, 2014; Reason, 2003; Shepherd-Barr, 1999, p. 157; Yeshurun & Sobel, 2010). In this case, the theatrical scent designer perhaps needs only to acknowledge, and thereafter to accept, the fact that the memories/association that are going to be triggered by a historically relevant scent are likely to be highly idiosyncratic (cf. van Campen, 2014; Verbeek & van Campen, 2013) and may well be imbued with personal meaning (especially when the scent is not grounded in a particular context; Slagle, 2014).

### Interim Summary: On the Multiple Uses of Deliberately Introduced Scent

Early attempts to introduce multiple, often abstract, scents in Symbolist performances and as an aid to (or means of) storytelling resolutely failed (e.g., Hartmann, 1913; Roinard et al., 1976). More successful have been the use of atmospheric/ambient scents that are very obviously linked to the action that is taking place on stage. In fact, according to Banes (2001), the use of scent has largely been illustrative (be it of words, characters, places or actions). However, scent has, on occasion, also been used to evoke a particular mood or atmosphere (e.g., Leach, 1989, pp. 89–90; see also Feagin, 2018). In her review, Banes (2001) suggests several possible further uses of scent including the contrastive, memorative, ritual, and/or defamiliarizing (as summarized by Jones, 2006, p. 45). According to Dominique Paquet, though, *direct exhibition* was the main flaw in the early olfactory staging of live shows. In fact, Paquet concluded her 2004 book on olfactory practices in contemporary theatre:

By the pessimistic observation of a series of relative failures of the odorization of shows, both for technical reasons but also for artistic reasons, in particular the primacy, with a few exceptions, of the use of smell as an illustration of a scene, of a character, of an object. (as quoted in Paquet, 2018, p. 86)

Scent has been used to try and create a specific mood and/or to try and trigger particular memories and/or a sense of nostalgia (Drobnick, 2009). At the same time, however, the simplistic use of scent has often been criticized. According to commentators, such as the influential Sally Banes, the pleonastic use of scent really does not serve much of a purpose in theatrical productions (Banes, 2001). Here, though, it is worth noting that many of the latest findings in the field of multisensory perception research can be taken to suggest that even when redundant, stimulating more senses (at least when done in a congruent manner) can help to engage people on a rational, as well as emotional, level (see Spence, 2021). Incorporating scent may also provide an effective means of helping to strengthen people’s emotional memories for events (e.g., Lwin et al., 2010; Verbeek & Van Campen, 2013; Ward, 2014), and indeed, the close link between scent and emotion is often mentioned (cf. Feagin, 2018). As such, it can be argued that even the pleonastic (i.e., illustrative or redundant) use of scent may nevertheless still help to pack an emotional punch to what might well otherwise be an anosmic theatrical production (Paquet, 2004).

The smell of actors cooking, smoking, and/or applying perfume has long been an incidental feature of the olfactory environment in the theatre (see Feagin, 2018; Margolies, 2003; Reason, 2003). As has been made clear at several points throughout the text, the recognition by audiences that they are inhaling the same scented air as the actors can exert a psychological influence, drawing attention to the connection between the audience and the actors on stage—helping to break down what is sometimes referred to as the “fourth wall” (McGinley & McGinley, 2018; see also Dugan, 2009; Feagin, 2018; Sale, 2006). This is, though, something that only occurs in a live-performance setting (i.e., and not in response to the scenting of the cinema).

Finally, one other use of scent is its potential to serve an almost contrapuntal, or contrastive, function (see Banes, 2001). McGinley and McGinley (2018, p. 225) draw the analogy with the use of slow music playing on the radio on stage when a chaotic fight scene is taking place on stage, say. Noah Bremer, director of *Haunted Basement* at The Soap Factory in 2013 had the following to say: “We don’t always want to have a scene where somebody is being butchered and there is the smell of blood. Maybe we want the smell of lilacs. It’s weird and disorienting” (as quoted in McGinley & McGinley, 2018, p. 225). Another example of the contrastive use of scent comes from Hamburg-based company Trollwerk’s ‘multi-media concert’ *Velcro Sync I 10* presented in Edinburgh in 2001, as highlighted by Margolies (2003). According to the programme notes: “through subtle overlaying of different media, associations will be played against each other (like pictures of war, the scent of strawberries).” In this case, however, the audience’s task is to try and recognize the unity behind the seemingly opposed. According to Margolies (2003),

Director Rico-Frank Heidler referred to Bergson's Matter and Memory as the inspiration for the juxtaposition of cognition and sensory perception overlaid with memory: “It’s the same as in daily life, sitting in front of the tv, seeing the news and smelling something from the kitchen” (Heidler, 2001). (pp. 16–17)

## Problems With Bringing Scent to a Live-Performance Setting

A number of the criticisms of the use of scent in live performance that have been voiced in recent decades have also been made in relation to its use in the cinema (see Spence, 2020c, for a review). In this section, I want to review a number of these criticisms and assess the extent to which they also apply to the live-performance setting.

### Can the Deliberate Use of Scent Ever be Anything More Than a Gimmick?

One of the most common complaints about the use of scent in cinema (i.e., in the plastic arts) is that it is never anything more than a gimmick. This was certainly the view expressed by filmmaker John Waters when interviewed by Avery Gilbert (2008, p. 167; see also Crowther, 1959; Sebag-Montefiore, 2015). It is legitimate to wonder, therefore, whether the same criticism could also be levelled at smell’s use in the live-performance setting? Certainly, there were those reviewers who complained that the use of scratch and sniff in *Love for Three Oranges* was mere “gimmickry” (Reynolds, 1989). Meanwhile, the scene in the Irish troupe Barrabas’s 1997 production of The Whiteheaded Boy, where one of the characters held up a piece of bread to a patently fake fireplace and suddenly, magically, the smell of toast wafted through the theatre also feels rather gimmicky too (Marks, 1999). The latter example, does though, perhaps represent one of the relatively few playful occasions where the attention of the audience has been deliberately drawn to the “artifice” of the scent. Thus, while the criticism of scent’s use being gimmicky is sometimes made in the context of live performance, it would seem to be expressed rather less frequently, perhaps linked to the typically much more austere use of scent in such a setting when compared with the cinema. The emphasis on shared experienced in the live, as opposed to the cinematic setting, is presumably also relevant here, with incidental smells naturally being experienced by the audience in the case of one but not the other.

### Misinterpreting the Source Object/Associations for Scents

The problem of establishing the appropriate associations with scent is something that has long been recognized by both practitioners and commentators (e.g., Hartmann, 1913; Runciman, 1915). However, the challenge of ensuring the appropriate interpretation for scent may have become rather more challenging following the arrival of synthetic chemistry and the widespread perfuming of so many of our household products (that so often seem to smell of pine/citrus, etc.; e.g., Margolies, 2003; Mitchell et al., 1964). Illustrating the problem, Rupert Christiansen (1999), writing in *The Daily Telegraph,* noted of one production that “the supposedly oriental smells wafting into the auditorium remind me of nothing more than Johnson’s floor polish.” Ben Randle also highlighted the problem with the synthetic referents for smells when talking about a production *Lord of the Rings* that he had been involved in:

“ … we were trying to play around with something that could be ‘spring,’ and as soon as you go there, everything you can come up with has been in some urinal or some bathroom or some disinfectant or some baby wipe,” he explains. “Scent memory can be very strong. When you have a scent with any bit of reference to something like that, you’re not eliciting the appropriate response. Unless, of course, your scene is in a urinal.” (quoted in Blankenship, 2016)

“Cleaning odour” was also the response elicited in a 2015 production of *Sagittarius Ponderosa*, a play by M. J. Kaufman, at San Francisco’s New Conservatory Theatre Center (and later in New York). In this case, a pine scent had been released into the theatre as the protagonist approached a Ponderosa pine (see Blankenship, 2016). The inability to constrain the emotional association(s) that the members of the audience may have had with scents, note, is also a problem that stymied early attempts to incorporate abstract olfactory stimuli in a theatrical or musical setting (e.g., Hartmann, 1913; Runciman, 1915). What is particularly striking about the former case, though, is that one would have imagined that the visual cues on stage (e.g., the presence of a pine tree) should have helped to constrain the audience’s interpretation of the scent (as indeed suggested by McGinley & McGinley, 2018, p. 222) in a way that simply did not seem to happen. That is, the cleaning scent interpretation won out, for at least some of the audience. However, this may be as much due to the use of a synthetic pine scent, rather than the genuine pine smell, as would presumably have been associated with the pine needles that had been sprinkled across the stage in the 1917 production of *Tiger Rose,* mentioned earlier (see Banes, 2001). Of course, given that the majority of urban dwellers now spend an estimated 90% or more of their lives indoors (see Spence, 2020a), it may well be that the synthetic pine scent has become the more familiar association for the majority of us than the real thing (cf. Verbeek, 2018). The increased use of synthetic fragrance in enclosed spaces is, though, not without its health-related challenges, as we will see later.

The use of ambient scents in the cinema as well as in the museum setting has sometimes been criticized for smelling overly “synthetic” (see Keller, 2014; Spence, 2020b, 2020c). However, while a similar criticism has, on occasion, been made in relation to scent’s use in the context of live performance, it is worth noting that this has sometimes been intentional, as we saw earlier (e.g., see Brantley, 1996).^18^ It is also worth noting how professional scent designers/perfumers have increasingly been involved in the sourcing/creation of the appropriate ambient scents for various productions in recent decades (e.g., Fanuel, 2015; Foster, 2017; Gilbert, 2008; Greenfield, 1989). This presumably means that the odours created for the live-performance setting are going to stand a greater chance of conveying their intended meaning, whatever that might be. At the same time, however, this also provides a link back to the early perfume maker Eugène Rimmel’s fragrances designed (or should that be promoted) to help deodorize London theatres and other entertainment venues in the middle decades of the 19th century.

### Dealing With Sensory Dominance

The incorporation of scent into theatrical productions has sometimes occurred alongside a reduction in visual input. Indeed, a number of scented performances have taken place in near or total darkness, or else with the audience encouraged to wear blindfolds (e.g., see Banes, 2001; Feagin, 2018; Gilbert, 2008; McGinley & McGinley, 2018; Tommasini, 2009).^19^ In Le Théâtre La Rubrique’s 1993 production of *Cendres de Cailloux* by Daniel Danis, for instance, “the audience was put in darkness during most of the two hours’ performance. During the course of the play, the actors used [ … ] natural essences to recreate, through smell, the feeling of being in the forest of Northern Quebec” (Lavoie, 1999, as cited by Banes, 2001, p. 70). Meanwhile, Susan L. Feagin (2018) describes a performance of *Till Birnam Wood*, a 1-hour adaptation of *Macbeth* at the Philadelphia Fringe Festival in 2015, where the members of the audience were given blindfolds to wear to weaken the dominant visual sense (see Hutmacher, 2019, on the theme of visual dominance). In this case, Feagin experiences the scent of pine late in the performance and writes about how she interprets it, with her blindfold on, as signifying Birnam Wood (not the smell of toilet cleaner)!

A few years ago, I myself was lucky enough to be invited to the *Future of Storytelling* conference held annually in New York (see short video at: *Charles Spence – Sensploration (FoST 2016),* https://vimeo.com/170509976). One of the most profound experiences showcased there (at least for my money) came from a couple of Dutch artists/scientists and involved a pair of mortuary fridges. As one newspaper puts it, “Dutch scientists are recreating the deaths of some of the world’s most famous personalities by reconstructing their last moments using scents and sounds” (see Delaunay, 2014). The idea is to recreate the last 5 minutes in the life of four people whose deaths are etched into so many of our collective memories: JF Kennedy (1963), Diana, Princess of Wales (1997), Muammar Gaddafi (2011), and Whitney Houston (2012).

Given the choice of inhaling the sweet smell of Jacqueline Kennedy’s perfume,^20^ mingled with the scent of John F. Kennedy’s blood, or bath time with Whitney Houston, I opted to experience the latter’s last drug-fuelled moments. The diva died (the coroner ruled it accidental) in an upmarket Beverly Hills bathtub in February 2012 at 48 years of age. After lying down on the metal tray as instructed, I was slowly rolled into the mortuary fridge, whereupon the door was slammed shut with an unnerving click of the latch. I suddenly found myself alone in pitch darkness in an almost claustrophobically confined space. The dominant visual sense (what Feagin, 2018, earlier called the “bully in the sensorium”) having been effectively eliminated, I got to experience the preprogrammed sequence of sounds and associated scents. These included the sounds of splashing water and Houston’s voice; I first caught a whiff of generic hotel cleaner, followed by the olive oil that the singer used in her tub. Then, a strong chemical odour, similar to that of cocaine, fills the box, grabbing its occupant by the throat, followed by the sound of rushing water, the electric crackling of her hairdryer short-circuiting as it slipped into the water, and finally silence. I doubt that this experience would have had anything like the same impact had there been an accompanying visual (that, at least, possible for JFK’s final moments).

The claustrophobic and confined space inside the mortuary fridge, as well as being semantically appropriate, turns out to be perfect for delivering, under computer control, and then venting, the 10 or so smells, while at the same time avoiding the olfactory overload that had so often interfered with earlier audience’s enjoyment of multismell productions, be they staged in the theatre or the cinema (see Spence, 2020d, for a review of the latter). *The Last Five Minutes* was a truly remarkable experience, one that promises a totally new way of “smelling” old stories (though I suppose it is worth noting that it wasn’t a live performance as such). One can, of course, think of this as a 21st-century version of Carl Sadakichi Hartmann’s early attempt at olfactory storytelling back in 1902 with his *A Trip to Japan in Sixteen Minutes* (Hartmann, 1913).

### Challenges With Scent Delivery in the Theatre/Scent Distribution

One of the problems with the cinematic use of scent has been that it has often proved difficult to synchronize the release (or rather the audience’s awareness) of scent with the action taking place on screen. However, this may be rather less of a problem in the theatre/live-performance setting (though see Gilbert, 2008, mentioned earlier), both because of the slower transitions in the theatrical context (e.g., contrast the length of a scene, when compared with the short cuts that are more typical contemporary film). There has also been more of an emphasis on the atmospheric, rather than narrative, use of scent in the live-performance context, again reducing the need for perfectly synchronized scent delivery. In the past, the audience’s exposure to scent in a live-performance setting has often been incidental, for example, as resulting from the actors smoking, cooking, or applying perfume (Margolies, 2003; Reason, 2003), and/or from the use of olfactorily-redolent stage props—remember the sides of beef that were hung on stage in the 1888 production of Fernand Icres’s *The Butchers* (see Banes, 2001). More recently, though, a host of different techniques have, more or less successfully, been used to deliver scent (at more or less the appropriate moment)—everything from actors pumping out/spraying scents from the proscenium and balconies through electric fans/bladeless Dyson fans (e.g., Foster, 2017; Legro, 2013), as favoured by so-called aroma/perfume jockeys (e.g., D’Errico, 2018; McGinley & McGinley, 2018). Other innovative solutions in this space have included the use of cold-mist humidifiers (Slagle, 2014) through scent tubing direct to each and every seat (Tommasini, 2009) and, on at least one occasion, scratch-and-sniff cards (for both live and TV audiences; see also McGinley & McGinley, 2018, pp. 223–224; Salesse & Domisseck, 2015). Back in the day, David Belasco (1853–1951) also added scent in the ventilation system during certain of his early aromatic productions (see http://www.valentinetheatre.com/mural/bios/David_Belasco.html).

In Richard Jones’s staging of Prokofiev’s opera *The Love for Three Oranges* for the English National Opera in 1989, scratch-and-sniff cards, much like those used in John Waters’s, movie *Polyester* (see Waters, 1981), were used (Banes, 2001; see also Paterson, 2006). In this case, they were created by Givenchy, the luxury French fashion and perfume house (Greenfield, 1989). The opera was broadcast on television by the BBC on Boxing Day, 1989. The listener (circulation 60,000) distributed the cards in its Christmas issue for viewers to use at home (Henry, 1989). There were six different scents to be scratched at various points in the performance. Writing at the time, Reynolds (1989, p. 3) described the smells as “oranges, [ … ] ‘an exotic perfume,’ [ … ] and ‘a cross between bad eggs and body odour’ for the entrance of Farfello, a demon noted for his bad breath and wind” (see also Hone, 2006).

The Guggenheim Museum premier of *Green Aria: A Scent Opera* was “*described as a beguiling 30-minute work*” with scents (30 distinctively named fragrances) created by the French perfumer Christophe Laudamiel (Alter, 2009; Lubow, 2009; Tommasini, 2009). Given that it was estimated that it would take up to 50 seconds for the scents to spread through the auditorium (the Peter B. Lewis Theatre) if delivered from the stage (even using a giant fan), it was decided that every seat had its own “scent microphone”—basically a tube that audience could bring as close to their nose as they liked. At the start of this multisensory experience, 5 elements and 18 supporting characters were introduced. As each character was announced, the audience heard the music and smelled the scent associated with the character. For example, “Fire + Smoke had crinkling electronic sounds and a piercing, burnt-ash scent.” The audience were seated in near darkness, experiencing an abstract drama of sound and scent. While this sounds like an intriguing example of scent’s foregrounding, at least in the context of contemporary performance, the press coverage associated with the event clearly highlights the fact that it was as much a vehicle for Laudamiel and his moneyed backer to create some noise around their new scented entertainment company (Aeosphere, a “fragrance media” company), as anything else (see Alter, 2009). Note that the Green Aria was sponsored by Thierry Mugler, the fashion, fragrance, and design house. The company was soon wound up though when the technology failed to materialize as planned (see https://www.casemine.com/judgement/us/5914e5c5add7b0493490879a).

Along similar lines to the *Green Aria,* D’Errico (2018) describes a production of *Les Parfums de l’Âme* by French playwright Violaine de Carné in a theatre that was suffused with incense while listening to the background sound of a requiem. In this case, a further 12 odours were diffused, each being linked to a specific character, or to one of their memories. Questionnaires completed by more than 300 of those who had attended one of these performances (as well as 35 individual interviews) indicated that the majority of people (77%) were satisfied with the olfactory element of proceedings—both the odour experience and the odour release system (Salesse & Domisseck, 2015).

In closing, it is noticeable, how a number of the most recent productions (e.g., *Green Aria: A Scent Opera, The Last Five Minutes,* and *Les Parfums de l’Âme*) have started to give olfaction a far more prominent role in the audience’s multisensory experience than has been the case since the very early days of scented theatre around the turn of the 19th century (e.g., Hartmann, 1913; Roinard et al., 1976). Tommasini (2009) captures this shift in emphasis when writing of the *Green Aria* that “the scents … became this opera’s characters.” While D’Errico (2018, pp. 234–235) talks of odour becoming a “theatrical object” and not just an illustrative or symbolic complement to a performance. Who knows, therefore, whether as the field of “aroma-turgy” (Blankenship, 2016) advances, it may become more common for live-performance audiences to be instructed not to wear perfume when going out to a show (Foster, 2017).^21^

### Multiple Chemical Sensitivities

It should, though, not be forgotten here that there have also been an increasing number of complaints in recent decades by those suffering from multiple chemical sensitivities, whose symptoms often appear to be triggered by the presence of synthetic ambient odour (see Spence, 2020e, for a review). No wonder, then, that some of those who have been working to introduce scent as a regular element in the context of theatrical performance have been minded to suggest that the audience should perhaps be warned, in advance, of any performance, that smells will be used (McGinley & McGinley, 2018, p. 225). This might start to seem like an eminently sensible precaution once it is realized that, according to the results of the latest multicountry survey, as many as one in three adults reportedly they suffer from a sensitivity to fragrance (Steinemann, 2019).

## Conclusions

There has always been a scent(-sory) component to people’s experience of the theatre. Initially, the smells would have been the possibly overpowering odour associated with the audience (their body odour, their food, and their effluence), the venue (the fusty air of the theatre) or, on occasion, of those incidental elements in proceedings (as when the actors cook, smoke, or apply perfume). At some point, though, the use of scent started to become more deliberate/intentional. First, it was used as an ambient (atmospheric) cue, and thereafter as a narrative element. On occasion, scent has also been used to convey a certain mood. While the early deliberate use of scent tended to involve nothing more exciting than its pleonastic use to match the setting/scene on stage, over the last half century or so, it has sometimes taken on a more pointed/political meaning. As we saw earlier, the sulphurous squibs (fireworks) that were let off at the start of Shakespeare’s Macbeth in the early 1600s likely had a polychromous meaning for the audiences of the day, referring not only to the action that was unfolding on stage but also to the recent Gunpowder Plot (see Harris, 2007). Ultimately, though, as McGinley and McGinley (2018) have noted recently, “Scent design should always add artistic value to a performance” (p. 224).

However, in contrast to the high-tech synchronized multisensory delivery of scent that one typically finds in the cinema (see Spence, 2020c, for a review), the live-performance setting has, with a few exceptions, tended to be low-tech. According to Sally Banes (2001), smell has been used in the theatre “to illustrate words, characters, places, and actions; to evoke a mood or ambience; to complement or contrast with aural/visual signs; to summon specific memories; to frame the performance as ritual; and to serve as a distancing device”^22^ (p. 69). Although, according to Banes (2001), most examples of smell in performance fall into the first two categories.

There can be little doubting the widespread interest in developing a more multisensory theatrical experience, be it involving immersive theatre or virtual reality (see Urwin, 2020, for a very recent example). The introduction of scent in the setting of the cinema, museum, and/or art gallery has, though, often not been successful, nor has it necessarily been positively received (see Spence, 2020b, 2020c, 2020d, for reviews). However, that being said, it is important to note that there is a profound difference between live performance and the cinema. In the live-performance setting, the performers and their audience experience the same sensory space, and hence the use of scent can help to break down barriers between them—this, the so-called fourth wall (e.g., Banes, 2001; McGinley & McGinley, 2018). By contrast, in the case of cinema, it is always clear to the audience that they cannot literally be smelling what the actors on screen are experiencing, and hence the experience of scent tends to break the narrative (and hence can all too easily seem gimmicky; see Spence, 2020c). This distinction between theatre and cinema (i.e., between the live and plastic arts) may be especially important given Sally Banes’ suggestion that the olfactory element of performance can potentially help to “carve out a niche for theatre where ‘liveness’ makes a difference” (Banes, 2001, p. 74). At the same time, however, it has been suggested that beyond any entertainment value, incorporating a scented element into live performance might also help increase accessibility for those with additional needs (Hawking, 2015).

## Notes

1 Here, it is worth noting that like many other forms of entertainment, the price of tickets for West End plays has been increasing rapidly in recent years, meaning that it is becoming too expensive for many normal people to see most popular productions (Ungoed-Thomas & Armstrong, 2019). Engaging more of the audience’s senses is often seen as one way of increasing the perceived value attached to an experience.

2 Unfortunately, the use of scent in non-Western performance lies beyond the scope of the present review. The interested reader is directed to Margolies (2003), Reason (2003), and Zgutowicz (1980).

3 Kirk-Smith (2003) documents the extensive appearance of the scent of lavender in early plays.

4 Note that flushing toilets first became widespread in the middle of the 19th century too (see Jütte, 2005, p. 171).

5 Jenner (2000, pp. 129–130) talks of the troubling “narrative of progress and deodorization” (see also Corbin, 1986).

6 All, note, pleasant and primarily floral odours (see Shepherd-Barr, 1999, p. 156, on this point).

7 According to Claude Schumacher (1996), “a bewildered audience was doused with perfume and left choking in fumes of incense which the ventilation system of the theatre was unable to cope with” (p. 18).

8 The outbreak of the First World War in Europe in 1914 likely changed people’s aesthetic inclinations/sensibilities too, hastening the demise of both Romanticism and Symbolist artistic tendencies (cf. Onion, 2018).

9 It is worth noting that varied associations and meanings triggered by the presentation of isolated olfactory stimuli were also highlighted as a problem by Runciman (1915) in his critical review of Scriabin’s desire to incorporate an abstract scented element into his 1911 *Prometheus: Poem of Fire* musical offering (Spence, 2020d).

10 According to one review of the proceedings,  Some found the whole thing preposterous and could not suppress their laughter; … others found it, even while recognizing certain problems, a sublime experience … The overall experience of scent was very different for individual spectators, depending upon how close they were to the vaporizers. (Deak, 1993, p. 155) Meanwhile, another reviewer suggested that the audience simply could not keep quiet because the release of the perfumes made them sneeze too much (as mentioned in Shepherd-Barr, 1999, p. 155).

11 Note here how the use of incense in churches also helps to mark the space out as “other” (Heffernan & Matter, 2001, p. 7).

12 While it is undoubtedly the case that olfactory experiences can sometimes be evoked in an almost synaesthetic manner by the characters on screen or stage (e.g., Jiaying, 2014; Reason, 2003), the experience is unlikely to be anything like as real when actually olfactory stimuli are presented.

13 Given that, as a child, I spend several years living over my parents’ fish and chips shop, Sunnybank Fisheries in Horsforth, Leeds, in the 1980s, I can all too easily imagine the smell, even some three decades later.

14 According to Greg Meeh, the president of J & M Special Effects, who has designed scents for a variety of shows (including the two just mentioned), “It’s a lot easier, and less expensive and time consuming, to deliver a subliminal aroma” (Blankenship, 2016). I am, though, a little unclear as to what exactly is meant by the term *subliminal aroma* in this case.

15 Here, it is perhaps worth noting that bad smells have been shown to lead to greater immersion in virtual environments than either positive or neutral smells (e.g., Baus & Bouchard; 2017; Ischer et al., 2014). The same may well also be true in a live-performance setting.

16 According to D’Errico (2018), in Peter Brook’s *Mahabharata* (1985), incense was burned throughout the play, thus providing an olfactory link both to the Indian context of the play, but also hinting at a more ritual connotation.

17 And that is before we get to Oscar Wilde’s purported desire to spray perfume throughout the auditorium for *Salomé* (Shepherd-Barr, 1999, p. 152).

18 See also Verbeek (2017, 2018) for early interest in specifically synthetic fragrances among the Italian Futurist artists such as Azari.

19 Although, of course, not all productions in the dark necessarily involve a scented element (e.g., Welton, 2007). In a sense, one might also consider this reminiscent of the dine in the dark restaurant concept (Spence & Piqueras-Fiszman, 2012, 2014).

20 Although the scientists had to use their imagination to recreate Jackie Kennedy’s perfume, as it is apparently no longer made.

21 At the same time, however, it is also worth noting that the smell of chemosignals emitted by the other members of the audience may add subtly to proceedings (D’Errico, 2018; see also Williams et al., 2016).

22 Banes (2001) also includes a seventh category, namely the ‘unrecognizable.’
